# Use of Machine Learning to Differentiate Children With Kawasaki Disease From Other Febrile Children in a Pediatric Emergency Department

**DOI:** 10.1001/jamanetworkopen.2023.7489

**Published:** 2023-04-11

**Authors:** Chih-Min Tsai, Chun-Hung Richard Lin, Ho-Chang Kuo, Fu-Jen Cheng, Hong-Ren Yu, Tsung-Chi Hung, Chuan-Sheng Hung, Chih-Ming Huang, Yu-Cheng Chu, Ying-Hsien Huang

**Affiliations:** 1 Department of Computer Science and Engineering, National Sun Yat-sen University, Kaohsiung, Taiwan; 2 Department of Pediatrics, Kaohsiung Chang Gung Memorial Hospital, Kaohsiung, Taiwan; 3 College of Medicine, Chang Gung University, Taoyuan, Taiwan; 4 Department of Emergency Medicine, Kaohsiung Chang Gung Memorial Hospital, Kaohsiung, Taiwan

## Abstract

**Importance:**

Early awareness of Kawasaki disease (KD) helps physicians administer appropriate therapy to prevent acquired heart disease in children. However, diagnosing KD is challenging and relies largely on subjective diagnosis criteria.

**Objective:**

To develop a prediction model using machine learning with objective parameters to differentiate children with KD from other febrile children.

**Design, Setting, and Participants:**

This diagnostic study included 74 641 febrile children younger than 5 years who were recruited from 4 hospitals, including 2 medical centers and 2 regional hospitals, between January 1, 2010, and December 31, 2019. Statistical analysis was performed from October 2021 to February 2023.

**Main Outcomes and Measures:**

Demographic data and laboratory values from electronic medical records, including complete blood cell count with differential, urinalysis, and biochemistry, were collected as possible parameters. The primary outcome was whether the febrile children fulfilled the diagnostic criteria of KD. The supervised eXtreme Gradient Boosting (XGBoost) machine learning method was applied to establish a prediction model. The confusion matrix and likelihood ratio were used to evaluate the performance of the prediction model.

**Results:**

This study included a total of 1142 patients with KD (mean [SD] age, 1.1 [0.8] years; 687 male patients [60.2%]) and 73 499 febrile children (mean [SD] age, 1.6 [1.4] years; 41 465 male patients [56.4%]) comprising the control group. The KD group was predominantly male (odds ratio, 1.79; 95% CI, 1.55-2.06) with younger age (mean difference, −0.6 years [95% CI, −0.6 to −0.5 years]) compared with the control group. The prediction model’s best performance in the testing set was able to achieve 92.5% sensitivity, 97.3% specificity, 34.5% positive predictive value, 99.9% negative predictive value, and a positive likelihood ratio of 34.0, which indicates outstanding performance. The area under the receiver operating characteristic curve of the prediction model was 0.980 (95% CI, 0.974-0.987).

**Conclusions and Relevance:**

This diagnostic study suggests that results of objective laboratory tests had the potential to be predictors of KD. Furthermore, these findings suggested that machine learning with XGBoost can help physicians differentiate children with KD from other febrile children in pediatric emergency departments with excellent sensitivity, specificity, and accuracy.

## Key Points

**Question:**

Can machine learning help physicians differentiate patients with Kawasaki disease from other febrile patients in a pediatric emergency department using only objective laboratory tests?

**Findings:**

In this diagnostic study that included 74 641 children with fever, the prediction model could identify Kawasaki disease with a sensitivity of 93% and a specificity of 97%.

**Meaning:**

These results suggest that machine learning can help physicians differentiate patients with Kawasaki disease from other febrile patients in a pediatric emergency department without relying on subjective symptoms.

## Teaser

This diagnostic study developed a prediction model using machine learning with objective parameters to differentiate children with Kawasaki disease from other febrile children.

## Introduction

Kawasaki disease (KD) is one of the most common forms of vasculitis in childhood and may cause acquired heart disease in children.^1,2^ Most morbidity and mortality associated with KD occur among patients with giant aneurysms.^3^ The incidence of coronary artery aneurysms decreased from 25% to about 4% if patients with KD were treated with intravenous immunoglobulin (IVIG) within the first 10 days of illness; as a result, the associated mortality was reduced.^2^ Therefore, early awareness of KD is vital for initiating IVIG therapy in time to minimize severe complications and prevent related mortality among children.

The diagnosis of KD is based mainly on clinical criteria. It requires the presence of fever lasting more than 5 days combined with at least 4 of the following 5 subjective physical findings: mucocutaneous inflammation, including bilateral nonexudative conjunctivitis; mucositis; polymorphous skin rash; extremity changes; and lymphadenopathy.^2^ Some children suspected of having KD but whose symptoms do not match the aforementioned epidemiologic case definition may have incomplete KD. These patients do not appear to differ from those with complete KD in any way except that they have fewer than 4 signs of mucocutaneous inflammation. Even among children with complete KD, these subjective signs of mucocutaneous findings are often not present simultaneously and have no standard order of appearance.^4,5,6^ All these factors make diagnosing KD challenging for first-line physicians evaluating febrile children in a pediatric emergency department (PED).

Some quick methods for the early identification of patients with KD have been proposed, but such methods are still far from being applied in clinical practice.^7,8,9^ Although no laboratory test values are included in the diagnostic criteria for complete KD, they may support making a KD diagnosis, particularly in incomplete cases.^2^ Hao et al^10^ proposed a classification tool for differentiating KD from other febrile illnesses using both subjective physical findings and objective laboratory test values. A novel score system to differentiate children with KD from febrile children using only objective laboratory test data was recently published.^11^ However, the positive likelihood ratio was only 5.11, indicating moderate evidence to rule in favor of KD. Therefore, more substantial evidence is warranted using only objective parameters to rule in favor of KD.

The advantages of machine learning approaches include their ability to process complex nonlinear associations between predictors and to yield more stable predictions.^12^ Over the past few decades, machine learning–based algorithms have been applied to many different fields and have attracted attention because of their superior ability to predict patient outcomes compared with traditional approaches in various settings and disease conditions.^13^ In recent years, machine learning has also demonstrated strong performance in assisting clinical diagnosis and outcome prediction in PEDs.^14,15,16,17^ Therefore, this study aimed to create a machine learning–based prediction model of KD using only objective laboratory tests to differentiate children with KD from other febrile children in the PED.

## Methods

### Study Population

Children with a diagnosis of complete or incomplete KD at the 4 main branch hospitals of Chung Gung Medical Foundation, Taiwan (2 medical centers in Linkou and Kaohsiung and 2 regional hospitals in Keelung and Chiayi), from January 1, 2010, to December 31, 2019, were evaluated for enrollment. All patients with KD were required to meet the diagnostic criteria stipulated by the American Heart Association (AHA).^2^ These children with KD were categorized as the KD group. Because KD rarely affects children of older age, most children in the KD group were younger than 5 years (98.6% [1142 of 1158]). Therefore, we enrolled febrile patients younger than 5 years without a diagnosis of KD as our febrile control (FC) group. Patients in the FC group presented to these 4 PEDs with fever during the same period as children in the KD group. Febrile children without laboratory test results were excluded from this study. This study followed the Standards for Reporting of Diagnostic Accuracy (STARD) reporting guideline for diagnostic studies. This study was approved by the Chang Gung Medical Foundation’s institutional review board, which also approved the waiver of the informed consent form because data were deidentified.

### Data Collection

Demographic characteristics (age and sex), date of visit, and laboratory test values, including urinary analysis with sediments, complete blood cell count with differential, C-reactive protein (CRP) level, aspartate aminotransferase level, and alanine aminotransferase (ALT) level, were collected from electronic medical records. Furthermore, medical notes describing symptoms and treatments were obtained from the electronic medical records to confirm the KD diagnosis. Pyuria may be seen in the urinalysis results of up to 80% of children with KD,^18^ indicating that it is an important feature. In this study, we applied 2 characteristics associated with pyuria. One is a categorical feature, pyuria or no pyuria, determined by the leukocyte esterase result in the urinalysis. The other characteristic is the white blood cell count in urine, which is a continuous variable that reflects the magnitude of pyuria. For children with KD who may have had repeated visits to the PED due to persistent fever or symptoms, a blood test may have been performed more than once, so we obtained the latest laboratory test results before the KD diagnosis was confirmed. All the aforementioned laboratory results could be obtained and evaluated by the physician at PEDs within 1 hour after obtaining the blood or urine samples. Because the result of a urinary culture takes at least 2 to 3 days, we did not use sterile pyuria as a feature in building our model. Erythrocyte sedimentation rate and albumin level are in the AHA list of supporting laboratory test data for KD diagnosis.^2^ Most febrile children lack these 2 laboratory test results in our PEDs, so we did not include them in our prediction model. If there were only 1 or 2 laboratory test results missing in each case, that item was filled with the mean value of the same laboratory item according to the group. If 3 or more laboratory results were missing, the case was excluded.

For the date of the PED visit, we performed one-hot encoding to transform it into categorical variables representing the month visited. For the other features, we did not do any preprocessing or feature engineering. The final data set was then used to perform the machine learning process.

### Model Establishment

Once obtained, all the cases were randomly split into the training set (80% of the cases) and the testing set (20% of the cases). There was no significant difference in variables between the training set and the testing set. We used Python programming language to build the prediction model. We chose the eXtreme Gradient Boosting (XGBoost) machine learning algorithm.^19^ Gradient boosting is a powerful ensemble method that combines base models in a sequential manner to achieve high predictive accuracy. In our study, we used imbalanced XGBoost^20^ for the classification of our KD data set, which is a highly label-imbalanced classification data set. Details of the hyperparameters used and imbalanced XGBoost are available in the eMethods in Supplement 1.

For a given data set *D* = {(*X_i_*, *y_i_*), *i* = 1, 2, 3, …, *N*, y ∈ (0, 1)} with *N* samples, the test data with *M* samples are denoted as *T* = {(*X_k_*, *y_k_*), *k* = 1, 2, 3, …, *M*, *y* ∈ (0, 1)}. A loss function is defined as *L*(*Y*, *Q*) = ∑*^M^_k_* _= 1_ *L*(*y_k_*, *q_k_*), where *y_k_* is the label (or outcome) and *q_k_* is the predictive value of some *X_k_* sample. We can define a binary classification problem as finding a function *f* that minimizes loss function *L*(*Y*, *f*(*X*)). XGBoost uses standard cross-entropy (*CE*) loss:



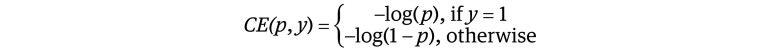



where *y* is the outcome that specifies the ground-truth class and *p* ∈ (0, 1) is the model’s estimated probability for the class with outcome *y* = 1. A common method for addressing class imbalance is to introduce a weighting factor α ∈ (0, 1) for class 1 and 1 − α for the other. That is, *CE*(*p_t_*) = −α log (*p_t_*). Facebook AI Research proposed another loss function, which is called the *focal loss*,^20^ to deal with the case of imbalance, in which a factor (1 − *p_t_*)^γ^ was added to the standard *CE* criterion: *FL*(*p_t_*) = −(1 − *p_t_*)^γ^ log (*p_t_*).

Setting the parameter γ > 0 reduces the relative loss for well-classified examples (*p_t_* > 0.5), focusing more on hard, misclassified examples. As our experiments for the KD data set demonstrate, the proposed focal loss enables training high performance when given a high recall (eg, 0.9) requirement condition.

### Statistical Analysis

Statistical analysis was performed from October 2021 to February 2023. We used the *t* test and the Fisher exact test or the χ^2^ test to compare clinical characteristics between children with and children without KD for continuous and categorical variables, respectively. Univariate and multivariate binary logistic regression analyses were used to identify significant risk factors. All *P* values were from 2-sided tests, and results were deemed statistically significant at *P* < .05. We used SPSS statistical software for Windows, version 22.0 (IBM Corp) for all statistical analyses.

## Results

### Patient Characteristics

**Figure 1.**
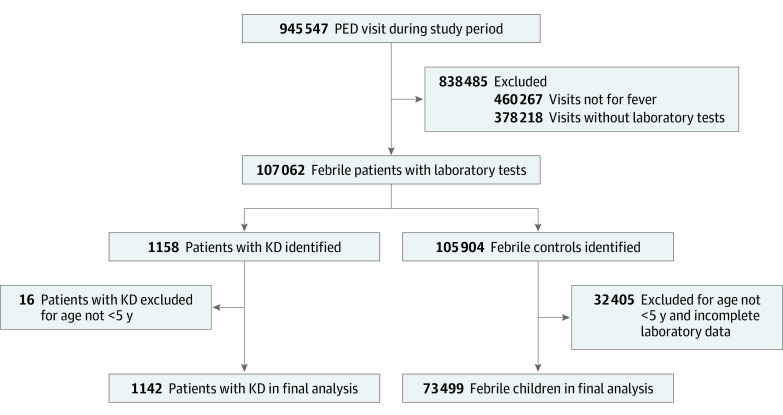
Selection Process of Patients With Kawasaki Disease (KD) and Febrile Controls PED indicates pediatric emergency department.

We identified a total of 1158 children with KD who had complete laboratory data during the study period. Among these children, 1142 (98.6%; mean [SD] age, 1.1 [0.8] years; 687 male patients [60.2%]) were younger than 5 years and were included to form our KD group. A total of 73 499 children (mean [SD] age, 1.6 [1.4] years; 41 465 male patients [56.4%]) younger than 5 years were identified to form our FC group. The flowchart of the patients’ selection process is shown in Figure 1. The sex, age, visit date, and laboratory test results were obtained from the electronic medical records for further analysis and were compared between the 2 groups. The KD group was predominantly male (odds ratio [OR], 1.79 [95% CI, 1.55-2.06]) with younger age (mean difference, −0.6 years [95% CI, −0.6 to −0.5 years]) compared with the FC group. The percentage of patients with pyuria was also higher in the KD group than in the FC group (523 [45.8%] vs 5324 [7.2%]; OR, 10.48 [95% CI, 8.98-12.24]). The other differences between the KD and FC groups are shown in Table 1.

**Table 1.** Basic Characteristics and Laboratory Test Values Between Children With KD and Febrile Controls

Characteristic	Children with KD (n = 1142)	Febrile controls (n = 73 499)	Odds ratio (95% CI)
**Categorical variables, No. (%)**			
Male sex	687 (60.2)	41 465 (56.4)	1.79 (1.55 to 2.06)
Pyuria	523 (45.8)	5324 (7.2)	10.48 (8.98 to 12.24)
Month of PED visit			
January	67 (5.9)	5419 (7.4)	0.783 (0.611 to 1.004)
February	62 (5.4)	4906 (6.7)	0.803 (0.620 to 1.038)
March	66 (5.8)	5931 (8.1)	0.699 (0.544 to 0.897)
April	101 (8.8)	6100 (8.3)	1.072 (0.872 to 1.317)
May	102 (8.9)	6644 (9.0)	0.987 (0.804 to 1.211)
June	105 (9.2)	6817 (9.3)	0.990 (0.809 to 1.212)
July	106 (9.3)	6801 (9.3)	1.003 (0.820 to 1.227)
August	145 (12.7)	6784 (9.2)	1.430 (1.199 to 1.705)
September	121 (10.6)	6424 (8.7)	1.237 (1.023 to 1.497)
October	101 (8.8)	6218 (8.5)	1.050 (0.854 to 1.290)
November	97 (8.5)	5858 (8.0)	1.072 (0.869 to 1.322)
December	69 (6.0)	5597 (7.6)	0.780 (0.611 to 0.997)
**Continuous variables, mean (SD)**			
Age, y	1.1 (0.8)	1.6 (1.4)	−0.6 (−0.6 to −0.5)^a^
WBC count, 10^3^/µL	14.1 (5.2)	10.9 (6.0)	3.3 (2.9 to 3.6)^a^
RBC count, 10^6^/µL	4.3 (0.5)	4.4 (0.6)	−0.09 (−0.13 to −0.06)^a^
Hemoglobin, g/dL	11.1 (1.1)	12.0 (1.8)	−0.8 (−1.0 to −0.7)^a^
Hematocrit, %	33.4 (3.1)	35.6 (5.0)	−2.2 (−2.5 to −1.9)^a^
MCH, pg/cell	25.7 (2.4)	27.2 (3.6)	−1.4 (−1.6 to −1.2)^a^
MCHC, g/dL	33.3 (1.1)	33.6 (1.2)	−0.3 (−0.4 to −0.2)^a^
RDW	13.4 (1.4)	14.1 (2.2)	−0.7 (−0.8 to −0.6)^a^
Platelets, 10^3^/µL	350.8 (125.3)	295.6 (126.5)	55.2 (47.8 to 62.6)^a^
Neutrophils–segments, %	56.1 (15.0)	48.4 (19.7)	7.7 (6.8 to 8.6)^a^
Neutrophils–bands, %	1.6 (3.2)	0.7 (2.1)	0.8 (0.7 to 1.0)^a^
Lymphocytes, %	32.3 (13.6)	39.0 (18.2)	−6.7 (−7.5 to −5.9)^a^
Monocytes, %	6.8 (3.4)	8.5 (4.3)	−1.6 (−1.9 to −1.4)^a^
Eosinophils, %	2.6 (2.7)	1.8 (2.9)	0.7 (0.6 to 0.9)^a^
Basophils, %	0.2 (0.4)	0.3 (0.5)	−0.04 (−0.06 to −0.02)^a^
AST, U/L	78.9 (120.0)	51.1 (118.1)	27.7 (20.7 to 34.7)^a^
ALT, U/L	76.8 (110.4)	34.7 (73.7)	42.1 (35.7 to 48.6)^a^
CRP, mg/dL	7.4 (6.0)	2.0 (3.2)	5.5 (5.1 to 5.8)^a^
WBC count in urine, count/hpf	55.0 (102.2)	50.2 (68.5)	4.86 (1.1 to 10.8)^a^

Abbreviations: ALT, alanine transaminase; AST, aspartate transaminase; CRP, C-reactive protein; KD, Kawasaki disease; MCH, mean corpuscular hemoglobin; MCHC, mean corpuscular hemoglobin concentration; PED, pediatric emergency department; RBC, red blood cell; RDW, red blood cell volume distribution width; WBC, white blood cell.

SI conversion factors: To convert WBCs to ×10^9^ per liter, multiply by 0.001; RBCs to ×10^12^ per liter, multiply by 1.0; hemoglobin to grams per liter, multiply by 10.0; hematocrit to proportion of 1.0, multiply by 0.01; MCHC to grams per liter, multiply by 10.0; platelets to ×10^9^ per liter, multiply by 1.0; neutrophils–segments, neutrophils–bands, lymphocytes, monocytes, eosinophils, and basophils to proportion of 1.0, multiply by 0.01; AST and ALT to microkatals per liter, multiply by 0.0167; and CRP to milligrams per liter, multiply by 10.0.

^a^ Mean difference (95% CI).

### Features Used to Predict KD

Among all categorical and continuous variables, pyuria, white blood cell count in urine, ALT level, CRP level, and eosinophil percentage were the top 5 features with the highest coefficients (18.01, 12.63, 7.65, 7.14, and 4.97, respectively) in the prediction model. Pyuria and the white blood cell count in urine were the only 2 features with coefficients greater than 10, indicating that pyuria found in urinalysis with sediment is an important feature for predicting KD. The other coefficients of features in our XGBoost prediction model are shown in Table 2.

**Table 2.** Coefficients of the eXtreme Gradient Boosting Prediction Model

Variable	Coefficient^a^
**Categorical variables**	
Male sex	1.06
Pyuria	18.01
Month of PED visit	
January	1.94
February	1.33
March	1.88
April	1.53
May	1.83
June	1.60
July	1.09
August	1.53
September	0.89
October	0.96
November	1.55
December	1.71
**Continuous variables**	
Age	2.77
WBC count	2.12
RBC count	1.39
Hemoglobin	1.13
Hematocrit	1.73
MCH	2.42
MCHC	1.10
RDW	2.16
Platelets	2.10
Neutrophils–segments	1.61
Neutrophils–bands	2.19
Lymphocyte	1.32
Monocyte	2.52
Eosinophil	4.97
Basophil	2.53
AST	3.54
ALT	7.65
CRP	7.14
WBC count in urine	12.63

Abbreviations: ALT, alanine transaminase; AST, aspartate transaminase; CRP, C-reactive protein; MCH, mean corpuscular hemoglobin; MCHC, mean corpuscular hemoglobin concentration; PED, pediatric emergency department; RBC, red blood cell; RDW, red blood cell volume distribution width; WBC, white blood cell.

^a^ The coefficient of these values was 100 times the originals, so the sum of all the coefficients equals 100, not 1.

### Model Performance

**Figure 2.**
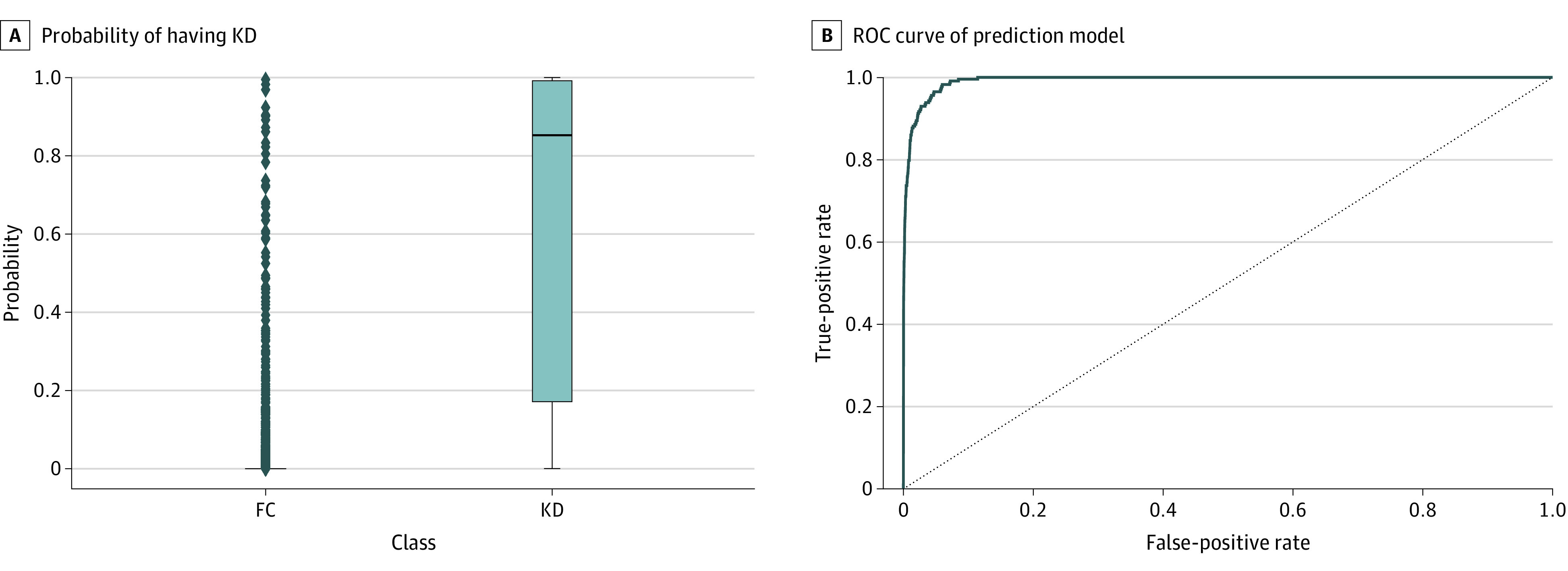
Probability of Having Kawasaki Disease (KD) and the Receiver Operating Characteristic (ROC) Curve of the eXtreme Gradient Boosting Model A, The quartile (Q)1, Q2, Q3, and IQR of the febrile control (FC) group are 0.000001, 0.000006, 0.000044, and 0.000001-0.000044, respectively. The Q1, Q2, Q3, and IQR of the KD group are 0.174867, 0.852702, 0.991781, and 0.174867-0.991781, respectively. Both Q1 and Q3 in the FC group are very close to zero, so the “box” is almost invisible. B. The area under the ROC curve is 0.980 (95% CI, 0.974-0.987).

To evaluate the performance of our prediction model, 20% of all data was preserved for validation, including 228 children with KD and 14 700 children in the FC group. After inputting the features in Table 2, the model yielded an output value between 0 and 1, which represented the probability of having KD. Figure 2A shows the probability of KD assigned by our model. We tried several different thresholds to test the overall performance of the model and to obtain a useful model for clinical settings. We wished to include as many cases as possible when screening patients with possible KD in the PED. Therefore, higher sensitivity is warranted with a lower threshold. As shown in Table 3, as we decreased the threshold to include more patients with KD, the sensitivity increased and the specificity decreased. Even though we lowered the threshold to achieve sensitivity greater than 90% (actual sensitivity was 92.5%), the specificity remained as high as 97.3%, with a 34.5% positive predictive value, a 99.9% negative predictive value, a high positive likelihood ratio of 34.0, and a low negative likelihood ratio of 0.08, indicating that our model has excellent performance. The area under the receiver operating characteristic curve (Figure 2B) of our prediction model was 0.980 (95% CI, 0.974-0.987). There were 101 children who received a new diagnosis of KD between 2020 and 2022 at our own hospital (Kaohsiung Chang Gung Memorial Hospital), and 93 of these children (92.1%) were correctly classified by our current model as having KD. The characteristics of children misclassified in the KD group and of children misclassified in the FC group are listed in eTable 1 in Supplement 1.

**Table 3.** Performance of the Prediction Model at Different Levels of Sensitivity

Variable	Target sensitivity, %					
	>70%	>75%	>80%	>85%	>90%	>95%
Sensitivity	71.1	75.4	84.7	86.0	92.5	100
Specificity	99.7	99.4	99.0	98.8	97.3	0.03
PPV	76.1	66.4	55.6	53.3	34.5	0.02
NPV	99.6	99.6	99.8	99.8	99.9	100
Positive LR	203.0	127.9	80.6	73.5	34.0	1.00
Negative LR	0.29	0.25	0.16	0.14	0.08	0.00

Abbreviations: LR, likelihood ratio; NPV, negative predictive value; PPV, positive predictive value.

### Subgroup Analysis

Because KD is at least a possible differential diagnosis for children with fever and elevated CRP level, we used 13 256 children in the FC group with a CRP level of more than 3 mg/dL (to convert to milligrams per liter, multiply by 10) and all 1142 children in the KD group to build 2 different prediction models: one used only the top 5 important features and the other used all features. The results still showed good sensitivity (95.2% in both models) and specificity (87.6% in the model using the top 5 features and 94.6% in the model using all features) in differentiating children with KD from those in the FC group. The performance of these 2 models is shown in eTable 2 in Supplement 1.

## Discussion

The main findings of this study were as follows: (1) the incidence of KD among febrile children was low; (2) pyuria, ALT level, CRP level, and eosinophilia were important features in predicting KD; and (3) a machine learning model established with XGBoost had an excellent ability to help physicians identify children with KD among all febrile children. The roughly estimated incidence of KD among febrile children was only approximately 1.5% (1142 of 74 641) in our cohort. In addition, 2 of our PEDs were in tertiary referral medical centers, where we accepted referrals from other hospitals for children with suspected KD. As a result, the proportion of children with KD among all febrile children is likely much lower than 1.5% in general clinical settings. Without pathognomonic tests, the diagnosis of KD continues to rely on identifying subjective clinical signs and excluding other clinically similar entities with known causes.^2^ Therefore, the challenge is to differentiate children with KD from febrile children when KD occurs at such a low rate. Reminding first-line physicians of the possibility of KD in febrile children is vital to prevent morbidity and mortality caused by coronary artery lesions. Hao et al^10^ developed a decision tree–based clinical algorithm to differentiate children with KD from other febrile children; the algorithm showed a good sensitivity of 96.0% and a specificity of 78.5%. Furthermore, their classifier achieved a sensitivity of 91.6% and specificity of 57.8% for multicenter validation in 2020.^21^ However, the subjective clinical signs associated with KD were also used as features in their prediction model. First-line physicians unfamiliar with these subjective signs of KD may lack awareness of this disease, limiting the use of the classifier developed by Hao et al.^10^

In the past several years, some quick and fancy methods, such as a wireless optical monitoring system,^7^ HAMP promoter hypomethylation,^8^ and proteome microarrays,^9^ have been proposed for the early identification of children with KD. These methods all use objective tests and parameters to help physicians diagnose KD, regardless of whether they are familiar with the subjective clinical signs. However, these methods lack validation and are still far from being applied in clinical practice. Recently, a new score system also tried to predict KD among febrile children by using only objective blood test results to reduce the reliance on subjective clinical presentation.^11^ Liu et al^22^ also established a nomogram model using only objective blood test results to discriminate children with KD from febrile children. Although the sensitivity could reach around 78% to 84% in validation, these 2 prediction models may still miss approximately 16% to 22% of cases of KD. Therefore, their utility in clinical practice may be limited. Compared with the previous score system^11^ and nomogram model,^22^ the biggest difference of our machine learning model is that we used urinalysis results, pyuria, and its magnitude as parts of our features, which we showed to have an important role in our model.

Kawasaki disease, a systemic vasculitis, can involve coronary arteries and multiple organs and tissues.^23^ Kidney manifestations in KD include pyuria, prerenal acute kidney injury, hemolytic uremic syndrome, immune complex–mediated nephropathy, acute kidney injury associated with KD shock syndrome, acute nephritic syndrome, nephrotic syndrome, and kidney tubular abnormalities. Of these kidney manifestations of KD, pyuria is the most common abnormal finding, which could be easily detected by urinalysis.^24^ In previous studies, the incidence of pyuria among children with KD has been reported as high as 60% to 80%, indicating its importance in children with KD.^25,26^ The reason why KD may cause pyuria is that the pyuria originates from the urethra, from the kidney due to mild and subclinical kidney injury, or from the bladder due to cystitis.^27^ Pyuria is also a suggested laboratory test listed by the AHA when evaluating the possibility of incomplete KD.^2^ Although a high prevalence of pyuria is found among children with KD, 1 previous study^25^ has demonstrated that the presence of pyuria was neither specific nor sensitive as a marker for KD. Nevertheless, in that study, the magnitude of pyuria was significantly higher among patients with KD when compared with children in the FC group. With these 2 important features of pyuria and its magnitude, our model can reach a higher performance than both the previous score system^11^ and the model developed by Liu et al.^22^

Because KD is a type of vasculitis, inflammatory biomarkers may help physicians with diagnoses. In evaluating the suspicion of incomplete KD, some objective laboratory test data were applied to support the KD diagnosis, including elevated CRP level, anemia for age, thrombocytosis, elevated ALT level, leukocytosis, and pyuria in urinalysis.^2^ In our study, we found that pyuria, elevated CRP level, and elevated ALT level were important features in predicting KD. One crucial feature not listed by the AHA but found to be significant in predicting KD by previous studies^11,22^ as well as our model is eosinophilia. Eosinophils are well known to play essential roles in type 2 inflammation, host immune defense, immunoregulation, and homeostasis.^28^ An early study showed that eosinophil levels were highly elevated in the acute phase of KD both before and after IVIG administration.^29^ Recent studies have also shown that immunoglobulin A (IgA) levels are associated with coronary artery lesions in KD^30^ and that eosinophils are required to generate and maintain mucosal IgA plasma cells.^31^ Chang et al^32^ also recently demonstrated the expression of eosinophilic subtype markers in children with KD. All of these findings support the critical role of eosinophils in children with KD.

### Limitations

Our study has some limitations. First, we included only children younger than 5 years, and they are all Taiwanese. Therefore, whether this model could also provide good prediction for older children or children of other races and ethnicities is unknown. Second, children with KD may have return visits to the PED and receive repeated laboratory tests. The laboratory test results that we used in the model were the most recent ones prior to diagnosis. Therefore, whether our model could be applied to earlier findings in tests to predict KD earlier is unknown. Third, a prospective study to fully validate the performance of this XGBoost model is warranted.

## Conclusions

This diagnostic study suggests that objective laboratory test results have potential as predictive parameters of KD. The prediction model established by the machine learning approach with XGBoost could help first-line physicians detect possible patients with KD among febrile children in a PED.

## Notes

Supplement 1. **eMethods.** Hyperparameters and Imbalanced XGBoost **eTable 1.** Characteristics of Children Misclassified as KD and Children Misclassified as FC **eTable 2.** The Performance of New Models in Subgroup Analysis at Different Levels of Sensitivity

Supplement 2.
Data Sharing Statement
