# Supplementary material for: Use of Machine Learning to Differentiate Children With Kawasaki Disease From Other Febrile Children in a Pediatric Emergency Department
Source: JAMA Netw Open. 2023 Apr 11;6(4):e237489. doi: 10.1001/jamanetworkopen.2023.7489 (PMC10091152; doi:10.1001/jamanetworkopen.2023.7489)
Supplement: Supplement 1. — eMethods. Hyperparameters and Imbalanced XGBoost eTable 1. Characteristics of Children Misclassified as KD and Children Misclassified as FC eTable 2. The Performance of New Models in Subgroup Analysis at Different Levels of Sensitivity [file jamanetwopen-e237489-s001.pdf]

Supplementary Online Content

Tsai CM, Lin CHR, Kuo HC, et al. Use of machine learning to differentiate children with Kawasaki disease from other febrile children in a pediatric emergency department. *JAMA Netw Open.* 2023;6(4):e237489. doi:10.1001/jamanetworkopen.2023.7489

eMethods. Hyperparameters and Imbalanced XGBoost
eTable 1. Characteristics of Children Misclassified as KD and Children Misclassified as FC
eTable 2. The Performance of New Models in Subgroup Analysis at Different Levels of Sensitivity

This supplementary material has been provided by the authors to give readers additional information about their work.

© 2023 Tsai CM et al. *JAMA Network Open.*

eMethods. Hyperparameters and Imbalanced XGBoost

Hyperparameters

For the Python XGBoost package we used, there are 5 hyperparameters, *max_depth, learning_rate (eta), booster, reg_alpha*, and *rea_lambda.* The booster we used is *gbtree*. In our experiment we only controlled both parameters *max_depth* and *learning_rate (eta)* which illustrated the best parameters from many different tests, and these two parameters were also used to control overfitting.

Imbalanced XGBoost

XGBoost, merited by the performance superiority and affordable time and memory complexities, has been widely applied to a variety of fields. However, XGBoost, like the other vanilla machine learning methods, suffers a performance decline if the ratio between labels becomes biased. Even though many experiments have showed that XGBoost can still outperform other methods on skewed datasets by being combined with other ensembling methods to achieve satisfactory results, this performance decay still leave significant negative effects on related research and applications. Therefore, XGBoost needs to be revised to ensure good performance in the task of imbalance.

© 2023 Tsai CM et al. *JAMA Network Open.*

eTable 1. Characteristics of Children Misclassified as KD and Children Misclassified as FC

Variables	Mean (SD)		Mean difference or odds ratios with 95%CI
	KD (misclassified as FC)	FC (misclassified as KD)	
	N=17	N=400	
Age (years)	1.34 (1.02)	1.18 (0.85)	1.21 (0.72 - 2.03)
Male Gender, N (%)	9 (52.9)	215 (53.8)	0.91 (0.33 - 2.53)
Pyuria, N (%)	5 (29.4)	134 (33.5)	0.84 (0.27 - 2.61)
WBC (10^3/uL)	9.91 (3.5)	13.8 (6.23)	3.9 (0.9 - 6.89)
RBC (10^6/uL)	4.43 (0.57)	4.42 (0.56)	-0.01 (-0.28 - 0.26)
Hemoglobin (g/dL)	11.77 (1.18)	11.26 (1.17)	-0.51 (-1.07 - 0.06)
Hematocrit (%)	34.92 (2.78)	33.82 (3.27)	-1.1 (-2.68 – 0.49)
MCH (pg/Cell)	26.69 (1.59)	25.68 (2.69)	-1.01 (-2.3 - 0.29)
MCHC (gHb/dL)	33.71 (1.27)	33.3 (1.07)	-0.41 (-0.93 - 0.11)
RDW	13.54 (1.06)	13.63 (1.94)	-0.09 (-0.84 - 1.02)
Platelets (10^3/uL)	316.06 (112.26)	322.46 (134.72)	6.4 (-58.79 - 71.59)
Segment (%)	52.55 (18.56)	50.36 (17.8)	-2.19 (-10.86 - 6.49)
Band (%)	0.53 (1.5)	0.87 (1.85)	0.33 (-0.56 - 1.23)
Lymphocyte (%)	36.34 (17.81)	38.03 (16.47)	1.68 (-6.36 - 9.73)
Monocyte (%)	7.62 (3.1)	7.94 (3.89)	0.32 (-1.56 - 2.2)
Eosinophil (%)	1.49 (2.4)	1.47 (2.09)	-0.03 (-1.05 - 1.0)
Basophil (%)	0.12 (0.25)	0.23 (0.38)	0.1 (-0.08 - 0.29)
AST (U/L)	54.11 (33.12)	55.27 (95.16)	1.15 (-44.38 - 46.68)
ALT (U/L)	46.06 (64.91)	40.81 (76.59)	-5.25 (-42.33 - 31.83)
CRP (mg/L)	25.94 (37.71)	48.54 (55.62)	22.6(-4.19 - 49.39)
U_{WBC} (count/hpf)	63.59 (132.76)	44.21 (106.0)	-19.38(-71.54 - 32.79)

Among 14929 FC in the testing set, 17 of 228 KD (7.5%) were misclassified as FC and 400 of 14701 FC (2.7%) were misclassified as KD .

© 2023 Tsai CM et al. *JAMA Network Open.*

eTable 2. The Performance of New Models in Subgroup Analysis at Different Levels of Sensitivity

Sensitivity target	>80%		>85%		>90%		>95%	
	Top-5	All	Top-5	All	Top-5	All	Top-5	All
Sensitivity (%)	81.6	81.6	85.1	85.1	90.4	90.4	95.2	95.2
Specificity (%)	95.2	99.1	92.8	98.3	90.6	97.0	87.6	94.6
PPV (%)	59.2	88.2	50.3	81.5	45.3	72.0	39.8	60.4
NPV (%)	98.4	98.4	98.6	98.7	99.1	99.2	99.5	99.6
+LR	16.9	86.5	11.8	51.3	9.6	30.0	7.7	17.8
-LR	0.2	0.19	0.2	0.15	0.1	0.1	0.05	0.05

In this subgroup analysis, those FC with CRP more than 30 mg/L (=3 mg/dL, this cut-off value is proposed in the evaluation of incomplete KD by AHA's guideline) were classified as New FC. Among original 73499 FC, 13256 FC with CRP more than 30mg/L and all 1142 KD were included in the subgroup analysis. Two new prediction model were built with the same strategy of the machine learning process as our original one. One model used only top-5 important features (CRP, ALT, urinalysis, and eosinophil count) as variables and the other one used all features. We could find that even if we exclude FC children with low CRP, the performance is still good. Besides, all features used in the model can enhance the model performance.

© 2023 Tsai CM et al. *JAMA Network Open.*
