# Supplementary material for: Use of Machine Learning to Differentiate Children With Kawasaki Disease From Other Febrile Children in a Pediatric Emergency Department
Source: JAMA Netw Open. 2023 Apr 11;6(4):e237489. doi: 10.1001/jamanetworkopen.2023.7489 (PMC10091152; doi:10.1001/jamanetworkopen.2023.7489)
Supplement: Supplement 2. — Data Sharing Statement [file jamanetwopen-e237489-s002.pdf]

Data Sharing Statement

Tsai. Use of Machine Learning to Differentiate Children With Kawasaki Disease From Other Febrile Children in a Pediatric Emergency Department. *JAMA Netw Open*. Published April 11, 2023. doi:10.1001/jamanetworkopen.2023.7489

Data
Data available: No
